# A novel endophytic species, *Streptomyces colwelliae* sp. nov., isolated from root nodule of *Alnus glutinosa*

**DOI:** 10.1186/s12866-025-04290-z

**Published:** 2025-09-18

**Authors:** Imen Nouioui, Juan Pablo Gomez-Escribano, Gabriele Pötter, Marlen Jando, Jacqueline Wolf, Meina Neumann-Schaal, Yvonne Mast

**Affiliations:** 1 Leibniz-Institut DSMZ – German Collection of Microorganisms and Cell Cultures, Inhoffenstraße 7B, Braunschweig, 38124 Germany; 2 Braunschweig Integrated Centre of Systems Biology (BRICS), Rebenring 56, Braunschweig, 38106 Germany; 3 Institut für Mikrobiologie, Technische Universität Braunschweig, Rebenring 56, Braunschweig, 38106 Germany

**Keywords:** *Actinomycetota*, Biodiversity, Taxonomy, Plant growth promoting bacteria, Agriculture

## Abstract

**Background:**

*Streptomyces* strains remain one of the most promising bacteria for novel drug discovery and are of general interest for pharmaceutical, biotechnological, and agricultural applications. They have been successfully used in agriculture to protect a number of crops, including wheat and rice, against fungal infections.

**Method and results:**

*Streptomyces* sp. Agncl-13^T^ was isolated from the surface sterilised root nodule of *Alnus glutinosa*, Newcastle upon Tyne, UK, and subjected to polyphasic taxonomic analysis and genome mining for plant growth promoting genes. Phenotypic, genetic and genomic data distinguished strain Agncl-13^T^ from *Streptomyces* species with validly published names. Whole cell hydrolysates of the strain were rich in *LL*-DAP. Strain Agncl-13^T^ had diphosphatidylglycerol, phosphatidylinositol, phosphatidylethanolamine, aminolipids, a glycolipid, a glycophospholipid, phospholipids and unidentified lipids, as polar lipids; as well as *iso*-C_15:0,_ *anteiso*-C_15:0_, *iso*-C_16:0_, C_16:0_, *iso*-C_17:0_, and *anteiso*-C_17:0_ as major fatty acids (> 5%), and MK-9(H_6_) and MK-9(H_4_) as predominant menaquinones (> 10%). Digital DNA-DNA hybridization values between the genomic sequence of strain Agncl-13^T^ and its phylogenomic relatives were below the threshold of 70%. The average nucleotide identity scores were below the thresholds 95–96% (bacteria) and 96.7% (*Streptomyces*) for bacteria species demarcation, respectively. The strain had several gene clusters whose products are involved in plant growth promotion. It was able to fix nitrogen, solubilise phosphate, and produce siderophore and ACC deaminase. In addition, strain Agncl-13^T^ contained several secondary metabolite biosynthetic gene clusters which had a low similarity confidence to known BCGs. The strain displayed antimicrobial activity against *Escherichia coli ΔtolC*,* Proteus vulgaris*, multidrug resistant *Staphylococcus aureus*, and *Candida albicans.*

**Conclusions:**

Based on polyphasic taxonomic data, the strain Agncl-13^T^ = DSM 118684^T^ = CECT 31215^T^ is considered as a novel species for which the name *Streptomyces colwelliae* sp. nov. is proposed. This study highlights the ecological features of strain Agncl-13^T^, which might be used as biocontrol agent and biofertilizer for sustainable agriculture. Further research investigations are needed to confirm the potential application of the proposed novel type strain.

**Supplementary Information:**

The online version contains supplementary material available at 10.1186/s12866-025-04290-z.

## Introduction

*Streptomyces* are filamentous bacteria with an extensively branched primary substrate mycelium, which subsequently develops into a secondary aerial mycelium with spores when cells reach maturity. The genus *Streptomyces*, belonging to the family *Streptomycetaceae* [1], the order *Kitasatosporales* [2], and the phylum *Actinomycetota*, contains more than 740 species with validly published names, with *Streptomyces albus* as the type species (https://lpsn.dsmz.de/genus/streptomyces) (access date 30.04.2025). These microorganisms are known for their saprophytic lifestyle, but they can also be endosymbionts and plant pathogens [2]. Streptomycetes are present in a wide range of environments, including extreme habitats. Their spore-forming ability makes them tolerant and resistant to various environmental stresses. They are characterised by the presence of a peptidoglycan cell wall, rich in *LL*-diaminopimelic acid, MK-9(H_6_) and/or MK-9(H_8_), and/or MK-10, and/or MK-8 as menaquinones; saturated *iso* and *anteiso* fatty acids, and diphosphatidylglycerol (DPG), phosphatidylethanolamine (PE), phosphatidylinositol (PI) and phosphatidylinositol mannosides (PIM), as predominant phospholipids [2]. The taxonomic status of this taxon has undergone several revisions due to advancements in phenotypic, genetic, and molecular approaches (chemotaxonomy, 16 S rRNA gene, multilocus sequence analysis (MLSA), DNA-DNA hybridization (DDH), G + C content) [3–5]. Modern prokaryotic systematics including genome sequence analysis and computational genomics (digital DDH (dDDH), average nucleotide identity (ANI), average amino acid identity (AAI), and percentage of conserved proteins (POCP)) have clarified the taxonomic relationships of heterogenous taxa and closely related species, and shifted the taxonomy to a more prominent level [6–8]. *Streptomyces* strains are renowned for their large genomes (> 8 Mb), which are rich in secondary metabolite-biosynthetic gene clusters (BGCs) encoding for specialised secondary metabolites [9], reflecting their ability to produce novel bioactive compounds. Two-thirds of all antibiotics in clinical use are derived from actinomycetes. In addition, several antitumor, antiparasitic, antifungal, and immunosuppressant agents have been derived from *Streptomyces* strains of various ecological niches [10]. *Streptomyces* remains one of the most promising bacterial genera for novel drug discovery, with applications in pharmaceutical, biotechnological, and agricultural fields [11, 12].

Furthermore, these microorganisms have been successfully employed in agriculture to protect various crops, including wheat and rice, from fungal infections [13, 14]. The ubiquity of *Streptomyces* in the soil and rhizosphere leads to extensive and direct interaction with the plant roots, where they are attracted by certain metabolites in root exudates and subsequently enter the endophytic compartments of the plant to compete for nutrients [15, 16]. Plant-endophytic streptomycetes are known for their ability to produce specialised metabolites that are beneficial to plant health, such as protection from pathogens and enhanced nutrient uptake such as *Streptomyces atrovirens*,* Streptomyces griseoviridis*,* Streptomyces lydicus*,* Streptomyces olivaceoviridis*,* Streptomyces rimosus*,* Streptomyces rochei*,* Streptomyces viridis* [17]. However, the plant growth-promoting capabilities of *Streptomyces* have only recently drawn attention [18]. Little is known about the influence of *Streptomyces* on plant nutrition (nutrient uptake) and their commercial availability as biocontrol agents remains limited. A small number of *Streptomyces-*based products are registered as biopesticide and biocontrol agents, including Actinovate^®^ (*Streptomyces lydicus* WYEC 108), Actofit^®^ and Astur^®^ (*Streptomyces avermitilis* MA-4680 and *Streptomyces avermitilis* MA-4848), Bialaphos^®^ (*Streptomyces hygroscopicus* SF-1293 and *Streptomyces viridochromogenes* Tü494), Incide SP^®^ and Actin^®^ (*Streptomyces atrovirens* DSM 41467^T^), Mycostop^®^ (*Streptomyces* sp. K61), Mykocide^®^ (*Streptomyces colombiensis* ATCC 27425), and Safegrow^®^ (*Streptomyces kasugaensis* BL904 and M338-M1) [17, 19–22]. Enhancing the genetic diversity of the genus *Streptomyces*, with a focus on plant-endophytic strains and their plant growth-promoting activities, is crucial for a sustainable agricultural system.

To this end, *Streptomyces* sp. Agncl-13^T^ (= DSM 118684^T^ = CECT 31215^T^) was isolated from the surface-sterilised root nodule of *Alnus glutinosa*, Newcastle upon Tyne, UK, and subjected to polyphasic taxonomic analysis and genome mining for plant growth-promoting (PGP) genes. The genetic ability of the strain to synthesise secondary metabolites was evaluated by computational analysis of the genome sequence for the abundance of BGCs and bioactivity of culture extracts of the strain has been shown with the help of bioassays. The results from the polyphasic taxonomic study confirmed that the strain represents a novel species, for which the name *Streptomyces colwelliae* sp. nov. is proposed. Additionally, the study highlights the biotechnological and ecological potential of the strain, suggesting its use as a biocontrol agent for sustainable agriculture.

## Materials and methods

### Ecological origin and isolation of strain Agncl-13^T^

Strain Agncl-13^T^ (= DSM 118684^T^ = CECT 31215^T^) was recovered from an *Alnus glutinosa* root nodule collected at Leazes Park, Newcastle upon Tyne, UK. Authorization to harvest root nodules has been given and this study complies with local and national regulations. The nodule was washed with water to remove soil and then surface sterilized using 30% H_2_O_2_, as described in Nouioui et al. [23]. A sterile lobe was mashed, suspended in 500 µl of saline solution (NaCl 0.9%), and incubated at room temperature for 10 min. One hundred microlitres of the supernatant was plated on BAP + agar medium (DSMZ 1536 (https://www.dsmz.de/microorganisms/medium/pdf/DSMZ_Medium1536.pdf)) and incubated for four weeks at 28 °C. A single colony of the strain was picked and cultured in DSMZ 65 medium (glucose (4 g/l) - yeast (4 g/l) - malt (10 g/l) extracts, calcium carbonate (2 g/l), agar (20/l), pH 7.2 for 14 days at 28 °C. The purity of the strain was confirmed using a light microscope (CARL Zeiss) and further validated through phenotypic and molecular analyses. The strain was deposited and maintained at the Leibniz-Institute DSMZ – German Collection of Microorganisms and Cell Cultures (DSMZ) and the Spanish Type Culture Collection (CECT). *Streptomyces prunicolor* DSM 40335^T^, the closest phylogenetic neighbour of strain Agncl-13^T^, was obtained from the DSMZ culture collection (https://www.dsmz.de/collection/catalogue) and included in all phenotypic, genetic and genomic analyses.

### Growth conditions, biochemical and enzymatic properties

Culture properties were determined after testing the ability of strain Agncl-13^T^ to grow on a wide range of agar media, including ISP1 to ISP7 (International *Streptomyces* project), nutrient agar (NA = DSMZ 1), GYM (Glucose-Yeast-Malt, DSMZ 65), TSA (Trypticase Soy Agar, DSMZ 535), and Bennett agar (DSMZ 548), under different pH values (5.0, 5.5, 6.0, 6.5, 7.0, 7.5, 8.0, 8.5, 9.0, 9.5), temperatures (4 °C, 10 °C, 15 °C, 20 °C, 25 °C, 28 °C, 37 °C, 42 °C, 45 °C), and salinity (0%, 2.5%, 5.0%, 7.5% NaCl). ISP2 agar medium was used for temperature, pH and salinity tests. Agar plates were inoculated with 20 µl of a bacterial suspension with a density of 5 on the McFarland scale [24]. The colour of the aerial and substrate mycelia of the strain was determined using the RAL colour chart. Spore chain morphology of the 7-day-old culture of the strain were examined using phase-contrast light microscopy (Nikon) at 100x magnification). The biochemical and enzymatic characteristics of the strain and its close neighbour, *S. prunicolor* DSM 40335^T^, were determined using the API 50CH, API 20 NE and API ZYM kits according to the manufacturer’s instructions (Biomérieux, France). All biomasses used for these tests were derived from 14-day-old cultures prepared in ISP2 medium incubated at 28 °C. All tests were performed in triplicate.

### Chemotaxonomic features

Strain Agncl-13^T^ and *S. prunicolor* DSM 40335^T^ were grown in ISP2 Liquid medium, shaken at 200 rpm for 10 days. The harvested biomasses were washed three times with a 0.9% saline solution and then freeze-dried. Whole cell hydrolysates were analysed for diaminopimelic acid [25]. Polar lipids of the strains were determined using two-dimensional thin layer chromatography as described by Minnikin et al. [26]. The isoprenoid menaquinones of the strains were extracted from fresh biomasses and identified using HPLC coupled to a diode array detector and high-resolution mass spectrometry, as described by Schumann et al. [27]. Fatty acid extracts were prepared following the protocol of Sasser [28] and identified by GC-MS on an Agilent GC-MS 7000D instrument [29]. The position of the single double bonds in the fatty acids was confirmed by derivatising the fatty acid methyl esters with dimethyl disulfide [30].

### Phylogenetic analysis, genome sequencing, and comparative genomic approaches

Genomic DNA, PCR amplification, and sequencing of the 16S rRNA gene of strain Agncl-13^T^ were conducted by the microbial DNA service at the DSMZ using a 96-capillary Applied Biosystems (ABI) [31]. The 16S rRNA gene sequence of the strain was compared with those of the type strain of *Streptomyces* species with validly published names using the EZBioCloud server (https://www.ezbiocloud.net/) [32]. Phylogenetic analysis based on Maximum-Likelihood (ML) and Maximum-Parsimony (MP) trees was performed using the DSMZ single-gene phylogeny server available at Genome-to-Genome Distance Calculator (GGDC; https://ggdc.dsmz.de/phylogeny-service.php#), as described by Meier-Kolthoff et al. [33]. The type strain *Embleya scabrispora* KM-4927^T^ was included as an outgroup. The 16S rRNA gene sequence similarity values between the strain and their closest phylogenetic neighbours were determined via the DSMZ single-gene phylogeny server at GGDC. Neighbor-joining phylogenetic tree was constructed with MEGA X software [34] using Kimura 2-parameter method [35] and 1000 bootstrap replications.

The genomic DNA of strain Agncl-13^T^ was extracted from a fresh culture grown in 5 ml ISP2 for 10 days at 28 °C with shaking at 120 rpm by the MicrobesNG service (https://microbesng.com/). In brief, Illumina sequencing with 250 bp paired-end reads and 30X depth of coverage was used. Read mapping, assembly, and taxonomic classification were conducted using BWA mem [36, 37], SPAdes software [38], and the Kraken tool [39], respectively. Genome annotation was carried out through Rapid Annotation using the Subsystem Technology platform (RAST) [40, 41]. The draft genome sequence has been deposited in GenBank under accession number JBLTJC000000000. To confirm the authenticity of the strain, the 16S rRNA gene sequence extracted from the genomic sequence was aligned with the sequence obtained by PCR, using the Basic Local Alignment Search Tool (BLASTN) available on the NCBI web server (https://blast.ncbi.nlm.nih.gov/Blast.cgi) [42, 43].

The genome sequence quality of strain Agncl-13^T^, including completeness and contamination, was assessed using BUSCO version 5.4.4 [44]. Genomic features, such as genome size, G + C content, number of RNA sequences, N50, and the number of coding sequences were determined using the RAST server [40, 41]. Genome-based phylogeny was inferred via the Type (strain) genome server (TYGS) (https://tygs.dsmz.de/*)* as described by Meier -Kolthoff et al. (2022) [33] and Meier-Kolthoff and Göker (2019) [45]. The dDDH value between strain Agncl-13^T^ and its closest phylogenetic neighbour *S. prunicolor* DSM 40335^T^ was calculated via the TYGS web server using the recommended formula *d*_4_ [33, 46–49]. The ANI value between the strains was estimated using the ANI calculator tool available on the EZBioCloud server (https://www.ezbiocloud.net/tools/ani) [50].

### In vitro and in silico evaluation of the biotechnological potential of strain Agncl-13^T^

Crude extracts of strain Agncl-13^T^ and its close relative *S. prunicolor* DSM 40335^T^ were prepared from active cultures grown in 50 ml ISP2, NL19, NL800, and R5 media, for 10 days at 28 °C with shaking at 180 rpm, as described in Nouioui et al. [51, 52]. Briefly, 5 ml of bacterial culture was collected and mixed with 5 ml ethyl acetate (EtAc) to extract the organic compounds. The mixture was incubated at room temperature for 3 to 6 hours under rotation. The organic phase was separated after centrifugation at 5000 rpm for 10 min and dried in an evaporator (SP Genevac EZ-2, “Low BP” program). The crude extracts were dissolved in 250 µl of 50% methanol and tested, *using* disc diffusion method, for antimicrobial activity against Gram-negative bacteria (*Escherichia coli ΔtolC* JW5503-1 and *Proteus vulgaris* DSM 2140), Gram-positive bacteria (multi-resistant *Staphylococcus aureus* DSM 18827 and *Enterococcus faecium* DSM 20477^T^), and yeast (*Candida albicans* DSM 1386). *E. coli ΔtolC* JW5503-1 was grown in 5 ml nutrient broth medium for 24 h at 37 °C with shaking at 120 rpm. *E. coli ΔtolC* JW5503-1 was obtained from the *E. coli* Genetic Stock Center, Yale, USA (collection number: 11430). The other reference strains and their growth conditions are available in the DSMZ online catalogue (https://www.dsmz.de/collection/catalogue*). Two hundred microlitres of bacterial (*2.0 × 10^6^ CFU/ml) *and yeast (*2.0 × 10^5^ CFU/ml) *suspensions of the reference strains were use as inoculum. *Sterile filter paper discs (6 mm in diameter) were impregnated with 15 µl of the crude extract, and then placed onto agar plates (50 ml square Petri dishes) inoculated with the tested microorganisms. The plates were incubated for 24 h at 37 °C for bacteria and at 25 °C for yeast. The ability of the *Streptomyces* strains to produce an antimicrobial compound was assessed based on the formation of inhibition zones against the tested microorganisms listed above. The antimicrobial tests were carried out in three biological replicates.

The genome sequences of strain Agncl-13^T^ and *S. prunicolor* NBRC 13075^T^ were analyzed for BGCs associated with specialized secondary metabolite using antiSMASH version 8.0 with default setting [53].

### Plant growth-promoting features based on in silico and in vitro analyses

The genome sequences of strain Agncl-13^T^ and its close phylogenetic neighbour *S. prunicolor* NBRC 13075^T^ were analysed for the presence of plant growth-promoting genes using the PGPT-Pred tool, available on the PLaBAse (version 1.02) platform (https://plabase.cs.uni-tuebingen.de/pb/plabase.php) [54–56]. The blastp + hmmer approach with the PGPT ontology and Krona strict mode blastp + hmmer were employed for this analysis.

Nitrogen fixation [57], phosphate solubilisation [58], siderophore, and ACC (1-aminocyclopropane-1-carboxylic acid) deaminase production [59, 60] were tested as the plant growth-promoting potential of strain Agncl-13^T^. Biomasses, from a 14-day-old culture prepared in ISP2 Liquid medium incubated at 28 °C with shaking at 120 rpm, were harvested, washed three times with sterile saline solution (0.9% w/v), and then dissolved in 0.9% NaCl (w/v). For all tests, a homogenous bacterial suspension with a density of 5 on the McFarland scale was used.

## Results and discussion

Strain Agncl-13^T^ exhibited morphological, phenotypic, genetic, and genomic features consistent with the genus *Streptomyces* [2]. The strain grew well on GYM, ISP2, and TSA, while growth was poor to moderate on ISP1, ISP4, ISP6, TSA, and NA after 14 days of incubation at 28 °C. On ISP2 medium, the strain developed a grey aerial mycelium with a brown exo-pigment that diffused into the medium. Agncl-13^T^ was able to grow on GYM medium within a temperature range of 20–37 °C, pH 5–9, and 0-2.5% NaCl (w/v), with optimal growth observed at 28 °C, pH 6.0-7.5, and 0-2.5% NaCl. The strain showed rectus-flexibilis spore chains. The strain was distinguishable from its close neighbour, *S. prunicolor* DSM 40335^T^, by its ability to metabolize D-mannose and D-xylose, while *S. prunicolor* DSM 40335^T^ oxidised glycerol (Table 1). Whole-cell hydrolysates of both strains were rich in *LL*-DAP (Figure S1). The polar lipid profile of strain Agncl-13^T^ consisted of diphosphatidylglycerol (DPG), phosphatidylinositol (PI), phosphatidylethanolamine (PE), aminolipids, a glycolipid, a glycophospholipid, phospholipids, and unidentified lipids. The same polar lipid content was detected for the related strain DSM 

 40335^T^ (Figure S2). Major fatty acids (> 5%) of strain Agncl-13^T^ and *S. prunicolor* DSM 40335^T^ were *iso*-C_15:0,_ *anteiso*-C_15:0_, *iso*-C_16:0_, C_16:0_, *iso*-C_17:0_, and *anteiso*-C_17:0_, although strain DSM 40335^T^ contained a lower amount of *iso*-C_17:0_ (Table S1). The predominant menaquinone (≥ 10)% in both strains were MK-9(H_6_) and either MK-9(H_4_) for Agncl-13^T^ or MK-9(H_8_) for *S. prunicolor* DSM 40335^T^ (Table 1). In tendency, *S. prunicolor* DSM 40335^T^ showed a shift to menaquinones with a higher saturation.

**Table 1:** Phenotypic and genomic features of strain Agncl-13^T^ and its close phylogenomic neighbour *S. prunicolor* DSM 40335^T^

	Strain Agncl-13^T^	S. prunicolor DSM 40335 ^T^
**Biochemical traits**		
Amygdalin	(+)	-
D-Arabinose	-	(+)
D-Arabitol	+	(+)
Dulcitol	(+)	-
L-Fucose	+	(+)
Gentiobiose	+	(+)
Glycerol	-	+
D-Lyxose	(+)	-
D-Mannose	+	-
Methyl-a-D-glucopyranoside	(+)	-
Potassium 2-ketogluconate	-	(+)
Potassium 5-ketogluconate	(+)	-
D-Raffinose	+	(+)
L-Rhamnose	+	(+)
Salicin	(+)	-
D-Trehalose	(+)	-
D-Xylose	+	-
L-Xylose	(+)	-
**Enzymatic activities**		
Cystine arylamidase	+	(+)
Trypsin	+	(+)
α-Fucosidase	-	(+)
Trypsin	+	(+)
Valine arylamidase	+	(+)
**Chemotaxonomic features**		
Polar lipid profile	DPG, PI, PE, ALs, GL, GPL, PLs, Ls	DPG, PI, PE, ALs, GL, GPL, PL, Ls
Major fatty acids (> 5%)	*iso*-C_15:0,_ *anteiso*-C_15:0_, *iso*-C_16:0_, C_16:0_, *iso*-C_17:0_, and *anteiso*-C_**17:0**_	*iso*-C_15:0,_ *anteiso*-C_15:0_, *iso*-C_16:0_, C_16:0_, *iso*-C_17:0_, and *anteiso*-C_**17:0**_
Predominant menaquinone ( ≥ 10%)	MK-9(H_4_) (23%), MK-9(H_6_) (61%)	MK-9(H_4_) (10%), MK-9(H_6_) (49%), MK-9(H_8_) (35%)
Diaminopimelic acid	*LL*-DAP	*LL*-DAP
**Genomic properties**		
Genome size (Mb)	11.79	11.76
G + C content mol %	70.0	69.7
Number of coding sequence	11,589	11,324
Number of RNAs	84	78
Genome accession number	JBLTJC000000000	BARF00000000.1

*DPG* diphosphatidylglycerol, *PI* phosphatidylinositol, *PE* phosphatidylethanolamine, *ALs* aminolipids, *GL* glycolipid, *PLs* phospholipids, *Ls* unidentified lipids

(+) weak reaction; + positive reaction; - negative reaction. All strains showed a positive reaction for L-arabinose, D-ribose, D-glucose, D-fructose, inositol, D-mannitol, N-acetylglucosamine, esculin/ferric citrate, D-cellobiose, D-melibiose, starch (amidon), glycogen, potassium gluconate, alkaline phosphatase, esterase (C 4), esterase lipase (C 8), lipase (C 14), leucine arylamidase, α -chymotrypsin, acid phosphatase, naphthol-AS-BI-phosphohydrolase, α -galactosidase, ß-galactosidase, ß-glucuronidase, α -glucosidase, ß-glucosidase, N-acetyl-ß-glucosaminidase, α -mannosidase. All strains showed a negative reaction for erythritol, D-adonitol, methyl-ß-D-xylopyranoside, L-sorbose, D-sorbitol, methyl- α -D-mannopyranoside, D-maltose, D-lactose (bovine origin), D-saccharose (sucrose), inulin, D-melezitose, xylitol, D-turanose, D-tagatose, D-fucose, L-arabitol. All strains showed a weak reaction for D-galactose, arbutin.

**Fig. 1:**
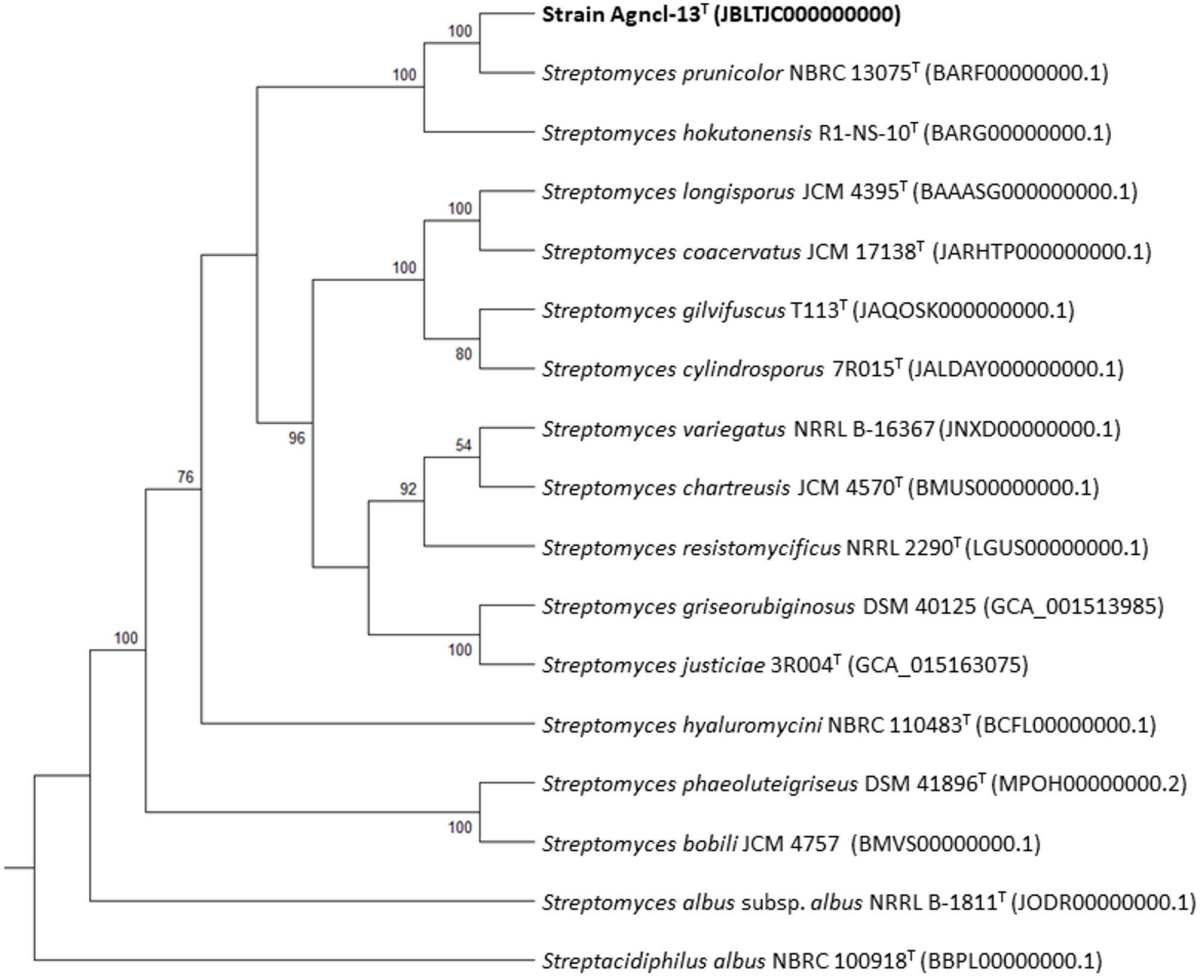
Tree inferred with FastME 2.1.6.1 from GBDP distances calculated from genome sequences. The branch lengths are scaled in terms of GBDP distance formula d5. The numbers above branches are GBDP pseudo-bootstrap support values > 50% from 100 replications

The nearly complete 16S rRNA gene sequence (1441 bp) of strain Agncl-13^T^, derived from PCR amplification, was identical to that extracted from the genome sequence, confirming the authenticity of the strain. The 16 S rRNA gene sequence similarity between strain Agncl-13^T^ and type strains of *Streptomyces* species with validly published names ranged from 98.4 to 99.9%, with *S. prunicolor* NBRC 13075^T^ being the closest phylogenetic neighbour (Table S2). The similarity with *S. prunicolor* NBRC 13075^T^ was 99.9%, well above the 98.65% threshold for prokaryotic species demarcation [61]. These results were in line with the phylogenetic position of Agncl-13^T^ in the ML, MP, and NJ trees (Figure S3 and S4), where the strain formed a well-supported sub-clade with the type strain of *S. prunicolor*, adjacent to the distinct branch of *S. hokutonensis* R1-NS-10^T^. The phylogenetic distance of the strain within the evolutionary radiation of the genus *Streptomyces* was further confirmed by its genome sequence, based on which strain Agncl-13^T^ and *S. prunicolor* formed a well-supported sub-clade (Fig. 1). dDDH values between the genomic sequences of strain Agncl-13^T^ and *Streptomyces* species, including *S. prunicolor* and *S. hokutonensis*, were below the 70% threshold for bacteria species delineation [62] (Table S3). These results were coherent with ANI values, which were below the defined threshold of 95–96% for bacterial species delineation and 96.7% for *Streptomyces* species demarcation (Table S3) [6, 63, 64].

### In vitro and in silico evaluation of the biotechnological potential of strain Agncl-13^T^

Strain Agncl-13^T^ has a genome size of 11.79 Mb, a G + C content of 70.0%, N50 of 24,498, 11,589 coding sequences, and 84 RNAs, characteristics that align with those typically found in the genus *Streptomyces* (Table 1). The genome sequence of Agncl-13^T^ had a BUSCO score of 97.8%, demonstrating a high level of completeness of the genome assembly (1544/1579 streptomycetales_odb10); however, the sequence quality is limited by a high degree of fragmentation, as reflected by the large number of contigs (> 1,000). Bioinformatic analysis using antiSMASH version 8.0 revealed that the draft genome sequences of Agncl-13^T^ and its closest phylogenetic neighbour *S. prunicolor* NBRC 13075^T^, contained a diverse set of secondary metabolite-BGCs, including clusters predicted to encode the biosynthesis of nonribosomally synthesized peptides (NRPs), ribosomally synthesized and post-translationally modified peptides (RiPPs), polyketides, terpenes, and alkaloids. A proper estimate on the BGC content could not be given as BGC detection is highly dependent on genome fragmentation and overall assembly quality, both of which were impaired in the Agncl-13^T^ genome sequence, leading to many fragmented BGCs. It was found that several of the detected BGCs were shared amongst the two strains, including those encoding flaviolin, desferrioxamine, scabichelin, ε-poly-L-lysine, and albaflavenone reflecting their phylogenetic relationship (Table S4). Furthermore, strain Agncl-13^T^ contained several unique BGCs not found in *S. prunicolor* NBRC 13075^T^, such as a phosphonate BGC with no detectable similarity to known clusters (Table S4).

The genetic machinery of strain Agncl-13^T^ was in line with its capacity to biosynthesise bioactive compounds with antimicrobial activity. The crude extract of strain Agncl-13^T^, prepared in DSMZ 65 medium, inhibited the growth of *E. coli ΔtolC* JW5503-1, *Proteus vulgaris* DSM 2140, methicillin-resistant *Staphylococcus aureus* DSM 18827 and *Candida albicans* DSM 1386. However, no antimicrobial activities were detected for *S. prunicolor* DSM 40335^T^ (Figure S5).

### Plant growth promoting features based on in silico and in vitro analyses

*Streptomyces* strains have recently been the subject of numerous studies related to probiotics and biocontrol agents for plants due to their ability to colonize plants, improve yield and protect crops from abiotic (heat, cold, drought, salinity) and biotic stresses. *Streptomyces* spp. are considered one of the most promising plant growth-promoting bacteria, offering an effective alternative to chemical pesticides and fertilizers, thus contributing to food security. Despite their potential, their commercialization as biocontrol and biofertilizing agents in sustainable agriculture remains limited, necessitating further research on plant-associated *Streptomyces.*

The endophytic lifestyle of strain Agncl-13^T^ along with the observed enrichment of BGCs potentially encoding specialised secondary metabolites points to a closer examination of the plant growth-promoting genomic characteristics of the strain. Genome mining of strain Agncl-13^T^ and *S. prunicolor* NBRC 13075^T^ revealed a broad range of BGCs whose potential products are involved in promoting and protecting plant growth (Table S5). Specifically, these strains possess the genetic machinery required to colonize plant systems (30% of PGP genes), compete for nutrient and adhesion sites (18% of PGP genes), improve soil fertility through phytohormone (11% of PGP genes) and biofertilizer (11% of PGP genes) production, act as stress biocontrol agents (20–21% of PGP genes), and function as bioremediators (10% of PGP genes). These findings highlight the beneficial ecological and agricultural potential of these strains.In silico data further suggest that these strains could be used as biofertilizers, as their genomes contain gene clusters associated with the acquisition of iron (Fe) and nitrogen (N), the solubilisation of phosphate and potassium (K), and the assimilation of sulfur (Table S5). These results were coherent within vitro tests, which confirmed the ability of strain Agncl-13^T^ to solubilize phosphate and fix nitrogen, as shown in Figure S6. Iron is a crucial micronutrient for plants, which can only assimilate it after Fe3^+^ is reduced to Fe^2+^ or by a chelation mechanism in the roots [65]. Insufficient iron uptake impairs plant growth and reduces crop yield. The lack of iron in the environment is usually related to its bioavailability rather than its low content. The same problem applies to phosphorus and potassium, which are essential elements for several biological processes in plants. Deficiencies in these elements can significantly reduce crop yields. Potassium plays an important role in the osmotic balance under abiotic and biotic stresses, assisting the plant in the uptake of nitrogen and phosphorus and improving CO_2_ absorption efficiency [66, 67]. Plant growth-promoting bacteria employ various mechanisms to acquire iron, solubilize inorganic P and K, and restrict sodium infiltration to restore the Na^+^/K^+^ ratio. These mechanisms include the production of siderophores, exopolysaccharides, organic acids, and hydroxyl ions [68–72]. All genes involved in such processes were found in the genomes of the studied strains, as detailed in Table S5. The ability of strain Agncl-13^T^ to produce siderophores was confirmed experimentally (Figure S6).

In addition, the genomic sequence of strain Agncl-13^T^ and *S. prunicolor* NBRC 13075^T^ contained BGCs associated with abscisic acid (ABA), cytokinines, and gamma-aminobutyric acid (GABA) production (Table S5). ABA promotes root elongation at its higher concentrations and protects the plant against drought and salt stresses by closing stomata to reduce water loss, while cytokinins stimulate stomatal opening, enhancing photosynthetic activity and thus promoting plant growth [73–75]. GABA is known to function as signaling molecule that helps to control environmental stress, as its accumulation has been linked to the reduction of reactive oxygen species (ROS) and, consequently improve plant tolerance to oxidative stress [76]. Furthermore, both strains contain genes encoding plant signaling molecules that positively impact plant germination and growth, and stress tolerance, particularly to drought, heavy metals, salinity, heat, and cold [77–80]. In vitro test confirmed the production of ACC deaminase by strain Agncl-13^T^ (Figure S6). In addition, the genomic sequence of these strains contained genes whose products are implicated in neutralizing osmotic, salinity, nitrosative, heat, and cold stresses, and biotic stress. Notably, the strains also harboured genes associated with the detoxification of heavy metals, such as antimony, arsenic, bismuth, cadmium, cobalt, copper, iron, lead, manganese, mercury, nickel, selenium, tellurium, and zinc, making them promising candidates for bioremediation. Furthermore, the strains exhibited several gene clusters related to colonisation and adaptation to the plant immune system, including those involved in plant cell wall membrane degradation, surface attachment, and root colonisation genes (Table S5). These results were consistent with the presence of fitness-associated genes, underlining the ability of the strains to withstand and survive in challenging environments, further highlighting their potential as biocontrol and biofertilizing agents. However, further research studies based on in vitro and *in planta* tests are required to confirm the results of the genome mining analysis and the potential application of the strain.

## Conclusion

Phenotypic, chemotaxonomic, genetic and genomic data differentiated strain Agncl-13^T^ from *Streptomyces* species with validly published names, supporting its classification as a novel species, for which we propose the name *Streptomyces colwelliae* sp. nov. Genome mining for plant growth-promoting associated genes revealed the genetic potential of the strain to be used as a biofertilizer and biocontrol agent. The results from antimicrobial bioassays further highlighted the antibiosis potential of the strain, indicating its effectiveness in preventing pests and inhibiting pathogens. This study emphasizes the need for further research into the plant growth-promoting properties of *Streptomyces*, which could have a beneficial impact on sustainable agriculture and the food system.

### Description of Streptomyces colwelliae sp. nov

*Streptomyces colwelliae* (col.well’i.ae N.L. gen. n. *colwelliae*, of Colwell, in honour of the American microbiologist Rita Rossi Colwell (University of Maryland, USA) for her significant contributions to marine microbiology and biotechnology).

Filamentous Gram-staining positive actinobacterium with rectus-flexibilis spore chains. Colonies have a white aerial mycelium on ISP2, ISP4, ISP7, and Bennett’s agar media after incubation of 14 days at 28 °C, and beige aerial mycelium on ISP3 and TSA agar plates. Optimal growth conditions were obtained on GYM, ISP2, ISP3, and Bennett’s media, pH 6.0-7.5, 0-2.5% NaCl, at 28 °C, and after incubation of 10–14 days. Metabolises D-arabitol, L-arabinose, D-cellobiose, D-fructose, L-fucose, gentiobiose, D-glucose, inositol, D-mannose, D-mannitol, D-melibiose D-raffinose, L-rhamnose, D-ribose, starch, and D-xylose, as sole carbon sources. Cells contain *LL*-DAP in their cell wall peptidoglycan; diphosphatidylglycerol (DPG); phosphatidylinositol (PI); phosphatidylethanolamine (PE), aminolipids, a glycolipid, a glycophospholipid, phospholipids and unidentified lipids as polar lipids; *iso*-C_15:0,_ *anteiso*-C_15:0_, *iso*-C_16:0_, C_16:0_, *iso*-C_17:0_, *anteiso*-C_**17**:0_ as the major fatty acids (> 5%); and MK-9(H_6_) and MK-9(H_4_) as the predominant menaquinone (> 10%).

The type strain Agncl-13^T^ (= DSM 118684^T^ = CECT 31215^T^) was isolated from surface sterilised root nodule of *Alnus glutinosa*, collected in Newcastle upon Tyne, UK. The strain has a genome size of 11.79 Mb and DNA G + C content of 70.0 mol%. The 16 S rRNA gene and genome sequences have been deposited in the GenBank under accession numbers PV124294.1 and JBLTJC000000000, respectively.

This Whole Genome Shotgun project has been deposited at DDBJ/ENA/GenBank under the accession JBLTJC000000000. The version described in this paper is version JBLTJC010000000.

## Supplementary Information

Supplementary Material 1.

Supplementary Material 2.

## Data Availability

The datasets generated and/or analysed during the current study are provided in the manuscript and as supplementary material. The 16 S rRNA gene and genome sequences of strain Agncl-13T have been deposited in the GenBank under accession numbers PV124294.1 and JBLTJC000000000, respectively.

## Abbreviations

ABA: Abscisic acid
ANI: Average nucleotide identity
BGCs: Biosynthetic gene clusters
BUSCO: Benchmarking Universal Single-Copy Orthologs
CECT: Spanish Type Culture Collection
dDDH: Digital DNA-DNA Hybridization
GABA: Gamma-aminobutyric acid
GBDP: Genome BLAST Distance Phylogeny
GC-MS: Gas Chromatography-Mass Spectrometry
ML: Maximum Likelihood
NRPs: Nonribosomal peptides
RiPP: Ribosomally synthesized and post-translationally modified peptide
ROS: Reactive oxygen species
