# Supplementary material for: A novel endophytic species, Streptomyces colwelliae sp. nov., isolated from root nodule of Alnus glutinosa
Source: BMC Microbiol. 2025 Sep 18;25:577. doi: 10.1186/s12866-025-04290-z (PMC12445025; doi:10.1186/s12866-025-04290-z)

**A novel endophytic species, *Streptomyces colwelliae*** **sp. nov., isolated from root nodule of *Alnus glutinosa***

Imen Nouioui^1*^, Juan-Pablo Escribano^1^, Gabriele Pötter^1^, Marlen Jando^1^, Jacqueline Wolf^1^, Meina Neumann-Schaal^1,2^, Yvonne Mast^1,2,3^

^1^Leibniz-Institut DSMZ – German Collection of Microorganisms and Cell Cultures, Inhoffenstraße 7B, 38124 Braunschweig, Germany.

^2^Braunschweig Integrated Centre of Systems Biology (BRICS), Rebenring 56, 38106 Braunschweig, Germany.

^3^Technische Universität Braunschweig, Institut für Mikrobiologie, Rebenring 56, 38106 Braunschweig, Germany.

*Corresponding author: Imen Nouioui imen.nouioui@dsmz.de


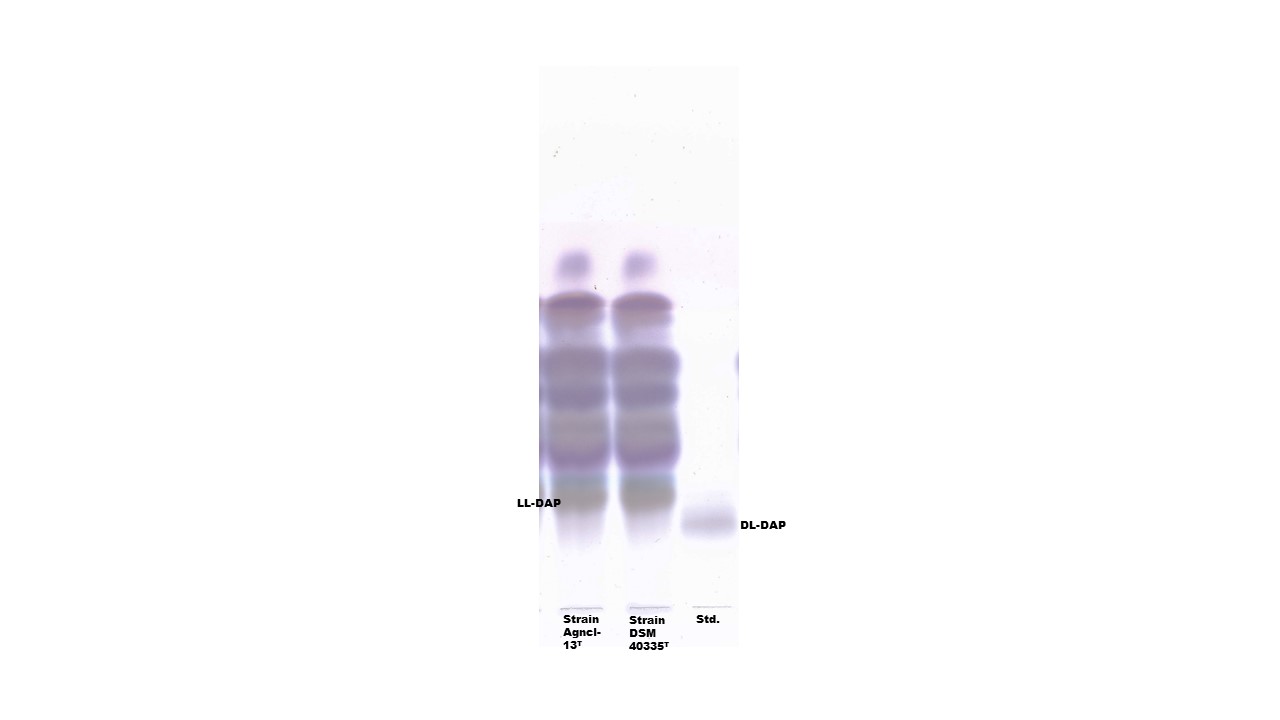


**Figure S1.** Diaminopimelic acid isomers of the whole cell hydrolysates of strain Agncl-13^T^ and its close relative *Streptomyces prunicolor* DSM 40335^T^.

**
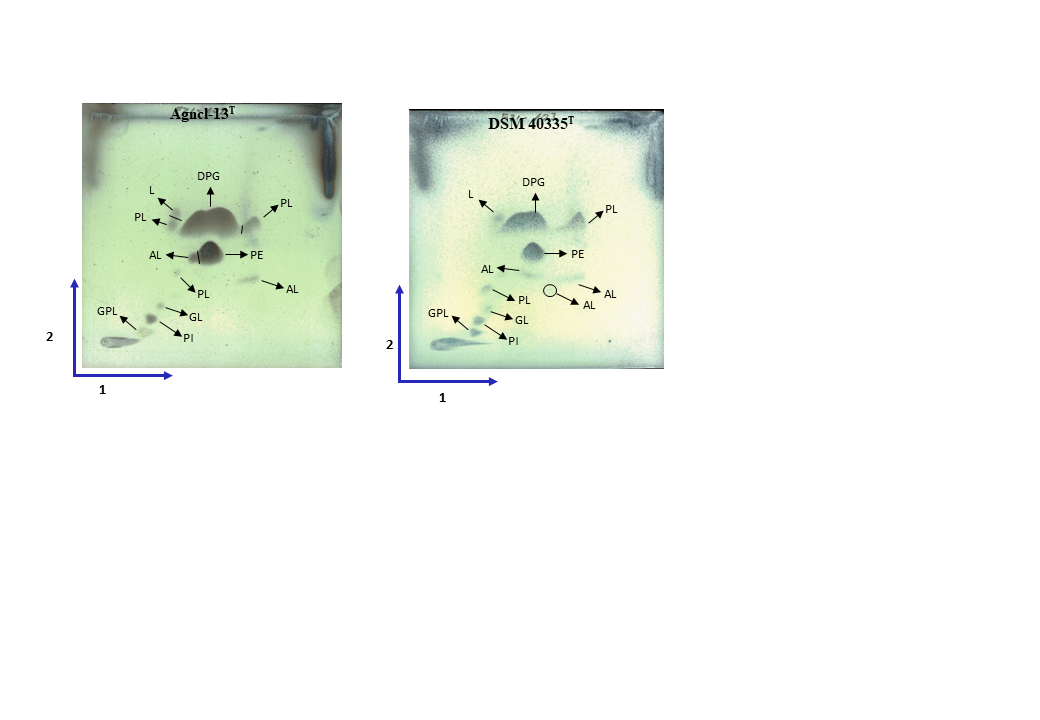
**

**Figure S2**. Two-dimensional TLC plate of polar lipids extracted from strain Agncl-13^T^ and its close phylogenomic neighbour *Streptomyces prunicolor* DSM 40335^T^ stained with molybdatophosphoric acid (Sigma P1518). Abbreviation: DPG, diphosphatidylglycerol; PI, phosphatidylinositol; PE, phosphatidylethanolamine; PL, phospholipid; L, lipid; GL, glycolipid; GPL, glycophospholipid, AL, aminolipid. Solvent 1: chloroform: methanol: distilled water (65:25:4 v/v/v); solvent 2: chloroform: glacial acetic acid: methanol: distilled water (80:12:15:4 v/v/v).

**
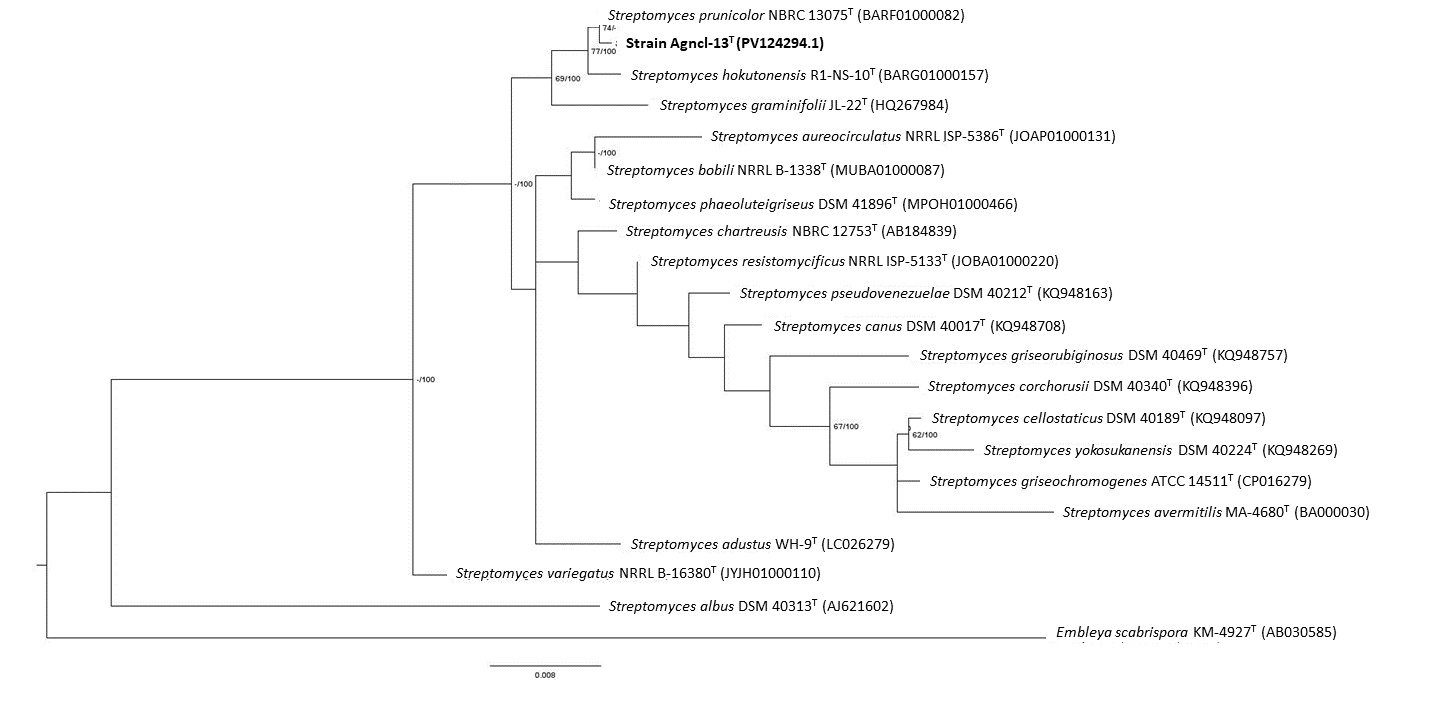
**

**Figure S3.** Maximum likelihood phylogenetic inferred under the GTR+GAMMA model and rooted by midpoint-rooting based on the 16S rRNA gene sequence showing the phylogenetic relationship of strain Agncl-13^T^ and its closest *Streptomyces* species with validly published names. The numbers above the branches are bootstrap values (> 60%) of ML (left) and MP (right).

**
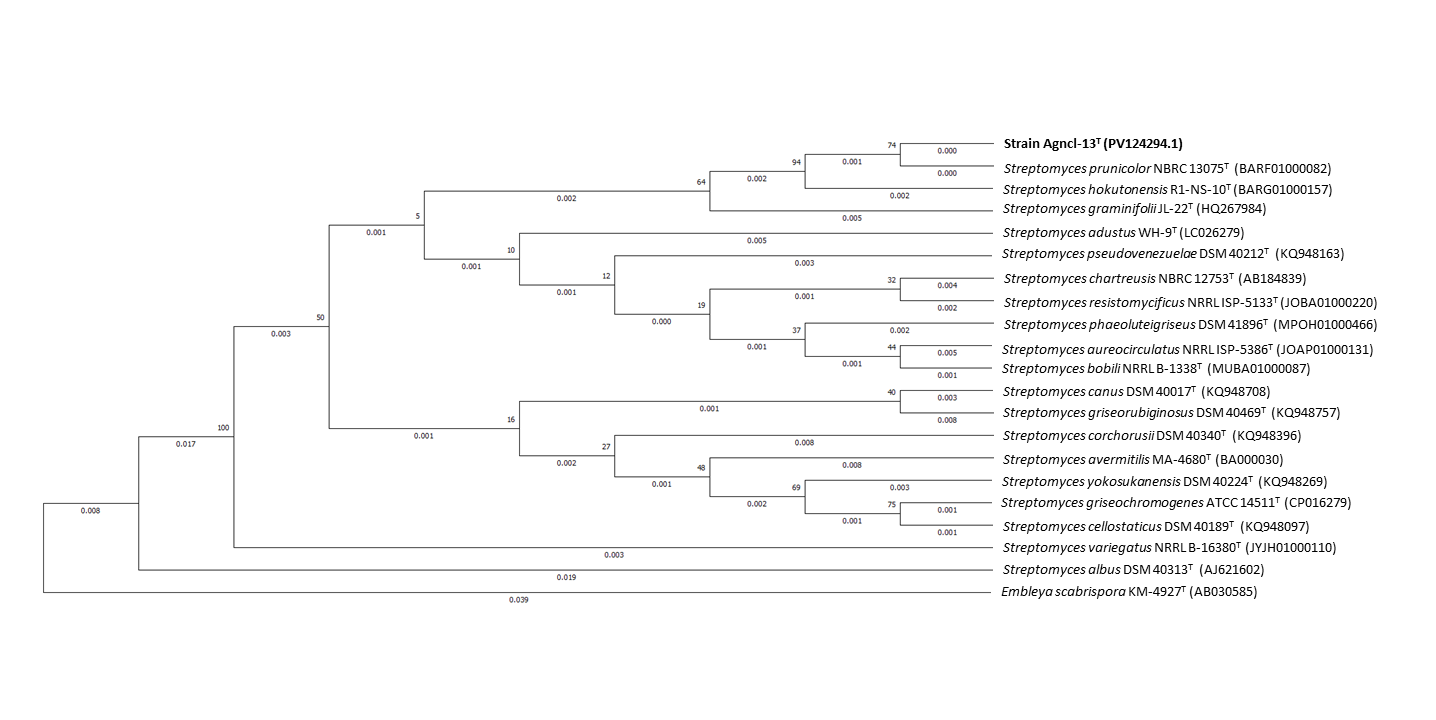
**

**Figure S4.** Neighbor-joining phylogenetic tree showing the phylogenetic relationship of strain Agncl-13^T^ within the evolutionary radiation of the genus *Streptomyces*. The numbers above and below the branches are bootstrap and branch length values, respectively.

**
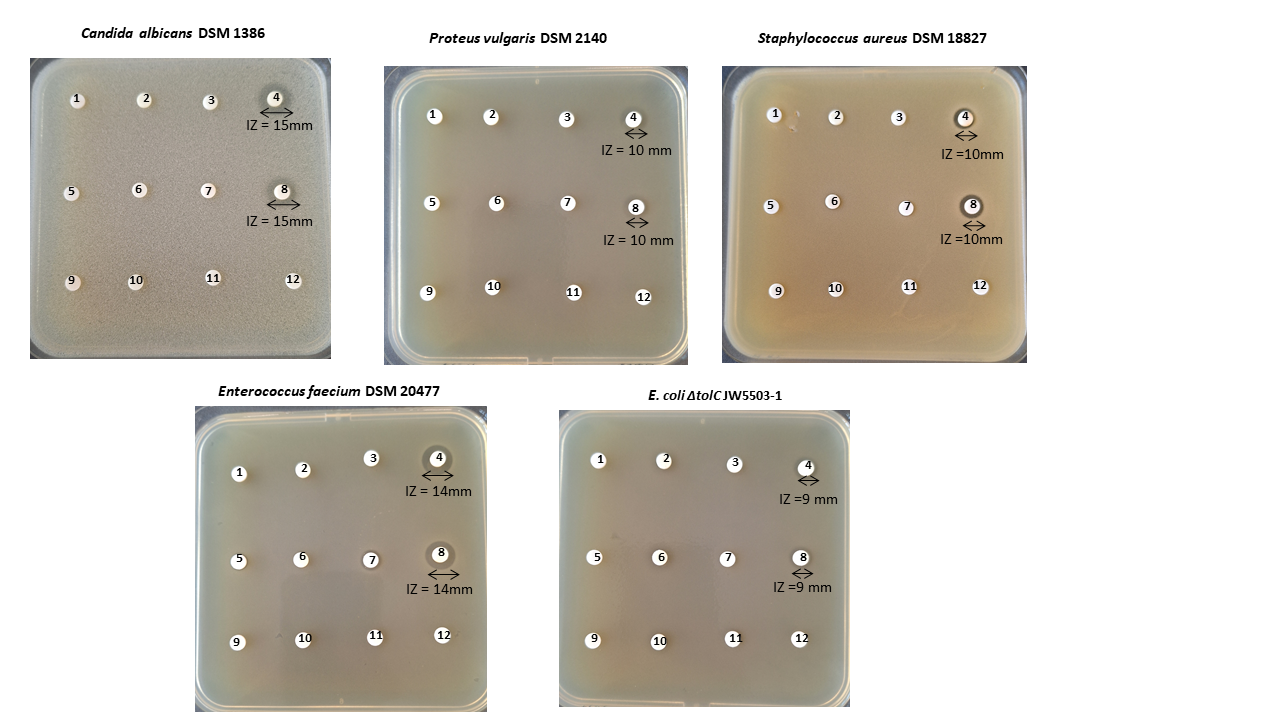
**

**Figure S5.** Antimicrobial bioassay of strains Agncl-13^T^ and *S. prunicolor* DSM 40335^T^ against *E. coli* *ΔtolC* JW5503-1, *Proteus vulgaris* DSM 2140, methicillin-resistant *Staphylococcus aureus* DSM 18827 and *Candida albicans* DSM 1386. Crude extract of strain Agncl-13**^T^** prepared in R5 (1, 5), NL19 (2, 6), NL 800 (3, 7), and DSMZ 65 (4, 8) media. Crude extract of strain DSM 40335^T^ prepared in R5 (9), NL19 (10), NL 800 (11), and DSMZ 65 (12) media. IZ = inhibition zone (mm).

**
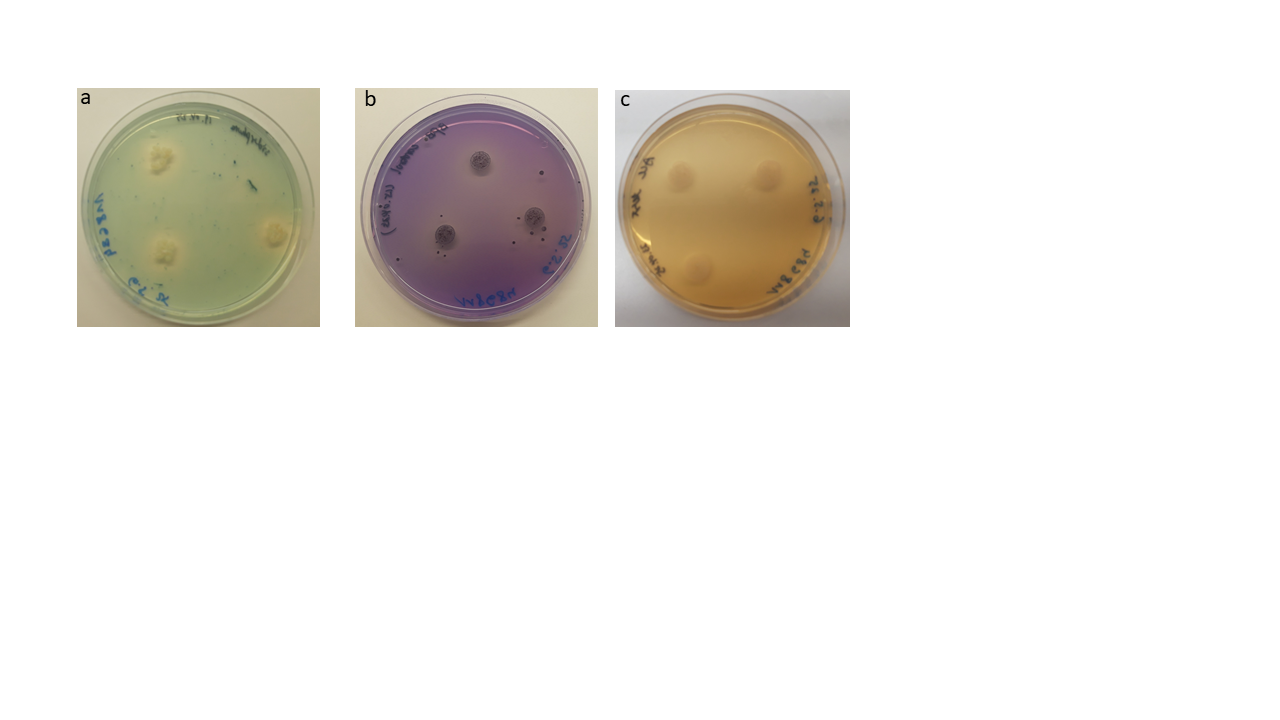
**

**Figure S6**. Production of siderophores (a) and ACC (1-aminocyclopropane-1-carboxylic acid) deaminase (c) and phosphate solubilisation (b) by strain Agncl-13^T^ after 7 days incubation at 28°C.

**Table S1.** Fatty acid profile of strain Agncl-13^T^ and its close phylogenomic neighbour *S.* *prunicolor* DSM 40335^T^

| **Fatty acid profile** | **Strain Agncl-13^T^** | **Strain DSM** **40335^T^** |
| --- | --- | --- |
| *iso*-C_14:0_ | 3.3 | 4.1 |
| *iso*-C_15:0_ | 12.4 | 9.0 |
| *anteiso*-C_15:0_ | 18.6 | 27.2 |
| C_15:0_ | 1.6 | 2.7 |
| C_16:0_ *cis* 9 | 5.0 | 1.6 |
| *iso*-C_16:0_ | 16.2 | 19.8 |
| C_16:0_ | 11.8 | 13.2 |
| *iso-*C_17:1_ *cis* 9 | 3.6 | 1.0 |
| *iso*-C_17:0_ | 8.9 | 3.8 |
| *anteiso*-C_17:0_ | 9.8 | 11.4 |

Only fatty acids above 2% are listed in the table.

**Table S2.** 16S rRNA gene sequence similarity between strain Agncl-13^T^ its close phylogenetic neighbours.

| **Reference type strains** | **Accession numbers** | **Similarity values (%)** |
| --- | --- | --- |
| *Streptomyces prunicolor* NBRC 13075^T^ | BARF01000082 | 99.9 |
| *Streptomyces* *hokutonensis* R1-NS-10^T^ | BARG01000157 | 99.7 |
| *Streptomyces graminifolii* JL-22^T^ | HQ267984 | 99.6 |
| *Streptomyces resistomycificus* NRRL ISP-5133^T^ | JOBA01000220 | 99.2 |
| *Streptomyces phaeoluteigriseus* DSM 41896^T^ | MPOH01000466 | 99.1 |
| *Streptomyces bobili* NRRL B-1338^T^ | MUBA01000087 | 98.9 |
| *Streptomyces chartreusis* NBRC 12753^T^ | AB184839 | 98.8 |
| *Streptomyces adustus* WH-9^T^ | LC026279 | 98.8 |
| *Streptomyces variegatus* NRRL B-16380^T^ | JYJH01000110 | 98.8 |
| *Streptomyces pseudovenezuelae* DSM 40212^T^ | KQ948163 | 98.7 |
| *Streptomyces canus* DSM 40017^T^ | KQ948708 | 98.7 |
| *Streptomyces griseorubiginosus* DSM 40469^T^ | KQ948757 | 98.7 |
| *Streptomyces aureocirculatus* NRRL ISP-5386^T^ | JOAP01000131 | 98.7 |
| *Streptomyces avermitilis* MA-4680^T^ | BA000030 | 98.7 |
| *Streptomyces corchorusii* DSM 40340^T^ | KQ948396 | 98.5 |
| *Streptomyces griseochromogenes* ATCC 14511^T^ | CP016279 | 98.5 |
| *Streptomyces cellostaticus* DSM 40189^T^ | KQ948097 | 98.5 |
| *Streptomyces yokosukanensis* DSM 40224^T^ | KQ948269 | 98.4 |

**Table S3.** Digital DNA-DNA hybridization (dDDH) and average nucleotide identity (ANI) between the whole genome sequence of strain Agncl-13^T^ and its close phylogenomic neighbours.

| **Subject strain** | **dDDH (%)** | **OrthoANI (%)** | **Genome accession numbers** |
| --- | --- | --- | --- |
| *Streptomyces* *prunicolor* NBRC 13075^T^ | 50.3 | 92.9 | BARF00000000.1 |
| *Streptomyces hokutonensis* R1-NS-10^T^ | 41.2 | 90.4 | BARG00000000.1 |
| *Streptomyces coacervatus* JCM 17138^T^ | 27.7 | 83.7 | JARHTP000000000.1 |
| *Streptomyces longisporus* JCM 4395^T^ | 27.6 | 83.6 | BAAASG000000000.1 |
| *Streptomyces resistomycificus* NRRL ISP-5133^T^ | 27.5 | 83.2 | JOBA00000000.1 |
| *Streptomyces cylindrosporus* 7R015^T^ | 27.3 | 83.5 | JALDAY000000000.1 |
| *Streptomyces resistomycificus* NRRL 2290^T^ | 27.2 | 83.2 | LGUS00000000.1 |
| *Streptomyces gilvifuscus* T113^T^ | 26.8 | 83.2 | JAQOSK000000000.1 |
| *Streptomyces justiciae* 3R004^T^ | 26.8 | 83.1 | JADDXU000000000.1 |
| *Streptomyces griseorubiginosus* DSM 40125^T^ | 26.6 | 82.7 | LMWK00000000.1 |
| *Streptomyces hyaluromycini* NBRC 110483^T^ | 26.3 | 82.8 | BCFL00000000.1 |
| *Streptomyces phaeoluteigriseus* DSM 41896^T^ | 26.2 | 82.4 | MPOH00000000.2 |
| *Streptomyces chartreusis* ATCC 14922^T^ | 26.2 | 82.7 | CP023689.1 |
| *Streptomyces chartreusis* JCM 4570^T^ | 26.2 | 82.6 | BMUS00000000.1 |
| *“Streptomyces humi”* MUSC 119^T^ | 26.0 | 82.4 | LBMU00000000.2 |
| *Streptomyces variegatus* NRRL B-16367 | 25.9 | 82.4 | JNXD00000000.1 |
| *“Streptomyces qaidamensis”* CGMCC 4.7315^T^ | 25.9 | 82.3 | CP015098.1 |
| *Streptomyces galilaeus* JCM 4757^T^ | 25.9 | 82.3 | BMVS00000000.1 |
| *Streptomyces albus* NRRL B-1811^T^ | 21.4 | 75.8 | JODR00000000.1 |
| *Streptacidiphilus albus* NBRC 100918^T^ | 20.8 | - | BBPL00000000.1 |

**Table S4**. AntiSMASH output for strains (A) *S. prunicolor* DSM 40335^T^ and (B) strain Agncl-13^T^. Colour code according to antiSMASH v. 7.0 (Blin et al., 2023).

A)
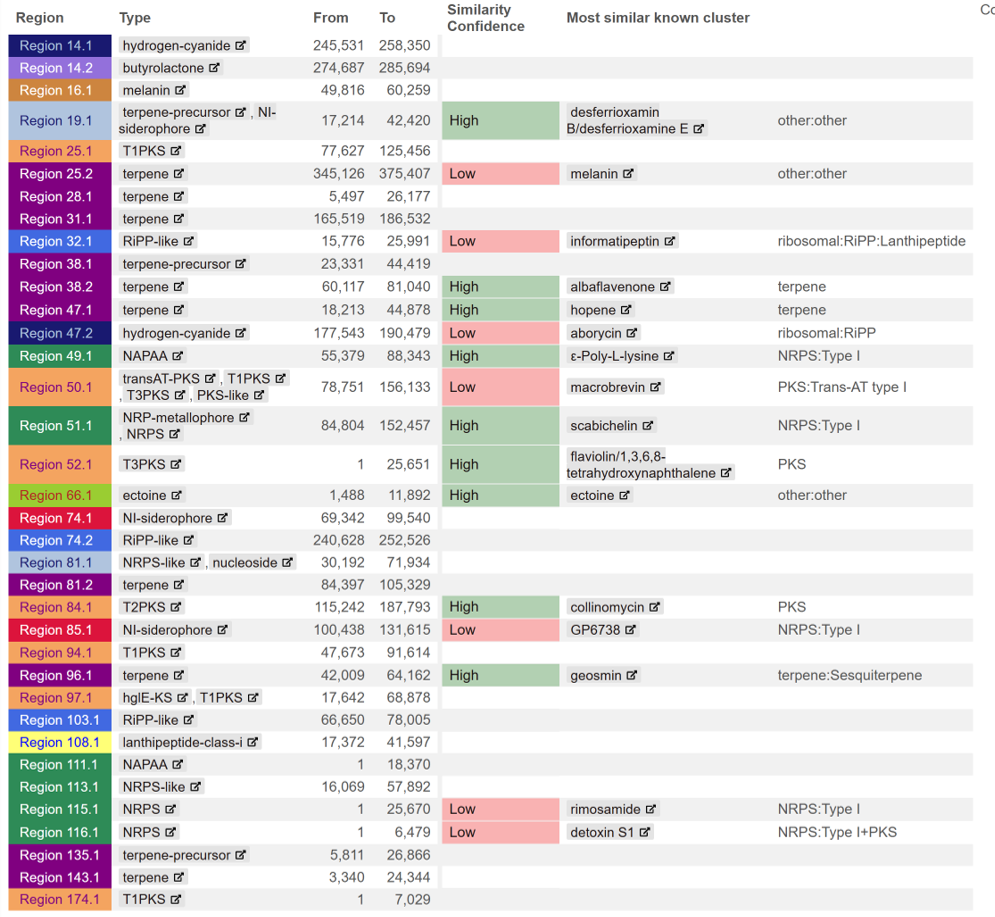


B)


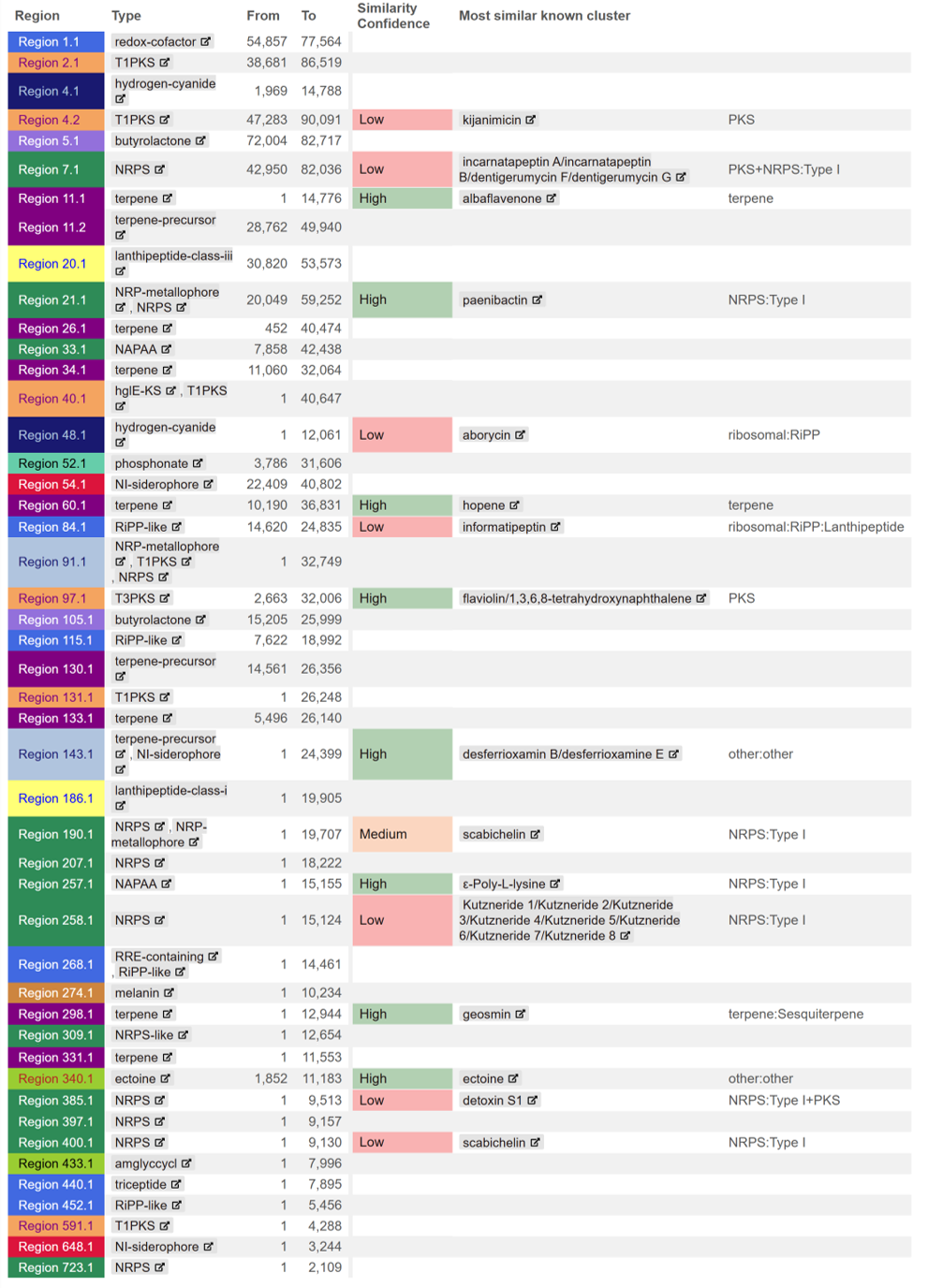

Supplement: Supplementary file 2 — Supplementary Material 2. [file 12866_2025_4290_MOESM2_ESM.docx]
